# Telephone-Based Reeducation of Drug Administration for *Helicobacterpylori* Eradication: A Multicenter Randomized Controlled Study

**DOI:** 10.1155/2020/8972473

**Published:** 2020-07-31

**Authors:** Yan Zhao, Mudan Ren, Xin Wang, Guifang Lu, Xinlan Lu, Dan Zhang, Shuixiang He

**Affiliations:** Department of Gastroenterology, First Affiliated Hospital of Xi'an Jiaotong University, Xi'an 710061, China

## Abstract

Poor adherence to treatment instructions may play an important role in the failure of *Helicobacter pylori* eradication. The aim of this study was to evaluate the effects of telephone-based reeducation on 14-day quadruple *H*. *pylori* eradication therapy. In total, 162 patients were randomly assigned (1 : 1) to either the intervention group (patients received telephone-based reeducation on the 4^th^, 7^th^, and 10^th^ days of the course) or the control group (patients received instructions only at the time of getting the prescriptions). All patients received a 14-day quadruple *H*. *pylori* eradication therapy. The primary outcome was the *H*. *pylori* eradication rate. The secondary outcomes included the symptom relief rates and the incidence rates of adverse events. Seventy-five patients in the reeducation group and 74 patients in the control group completed the follow-up. The *H*. *pylori* eradication rate in the reeducation group was statistically higher than that in the control group (intention-to-treat: 72.8% vs. 50.6%, *P* = 0.006; per-protocol: 78.7% vs. 55.4%, *P* = 0.003). However, the symptom relief rates and the adverse event rates in these two groups were not significantly different. Overall, the results from this study suggest that telephone-based reeducation can be potentially applied to improve the *H*. *pylori* eradication rate in clinical practice, without significantly increasing the adverse effects.

## 1. Introduction

*Helicobacter pylori* is highly prevalent worldwide [1]. It affects 50% of the world population, with an infection rate of 90% in developing countries [2]. It was reported that more than 50% of the Chinese population was infected with *H*. *pylori* in the past ten years and the infection rate even reached 70% in some areas [3]. *H*. *pylori* is considered a class I carcinogen for gastric carcinoma and mucosa-associated lymphoid tissue lymphoma and is associated with chronic gastritis, peptic ulcer disease, and other digestive diseases [4]. Eradication of *H*. *pylori* is strongly recommended because it could help promote ulcer healing, reduce ulcer recurrence, reduce the incidence of digestive malignancies, and thus improve patients' quality of life [5, 6]. However, *H*. *pylori* is a highly genetically and phenotypically adapted pathogen and the eradication success rate of the conventional triple therapy has decreased to 80% in many countries [7–9].

Although various pathogens and host-related factors have been reported to cause the failure of *H*. *pylori* eradication, antibiotic resistance and poor adherence are considered the two major factors that contribute to the failure of treatment [10, 11]. A study reported that the eradication rate was 96% in patients who finished at least 60% course of the antibiotic treatment, while successful eradication was observed in only 69% patients taking less than 60% of the prescriptions [12]. Moreover, during *H*. *pylori* eradication, poor adherence significantly resulted in treatment failure in antibiotic-sensitive patients [13]. Multiple factors, such as duration and complexity of the treatment, could affect the treatment adherence, especially in cases in which the prescribed dosage and administration are different from the drug label instructions. The prolonged 14-day eradication course and quadruple therapy, recommended by the latest guideline, may further aggravate the current situation [14]. Furthermore, the complexity of drug administration may affect compliance in patients belonging to low-income area, with relatively low educational background.

Due to the cost-effectiveness and convenience, text messages and phone calls have been used to improve health outcomes in several fields, such as preventative programs, disease management, and the improvement of adherence to medication [15, 16]. At the same time, they have been well accepted by users as an effective way for communicating healthcare information [17, 18]. A prospective study, which was based on the 10-day triple therapy, reported no significant effect of telephone-based reeducation on *H*. *pylori* eradication and patients' adherence to treatment [19]. However, the 14-day quadruple therapy was favored by the latest consensus and this longer treatment may aggravate the poor compliance, owing to prolonged duration and increased complexity [20, 21]. Therefore, we conducted this multicenter randomized controlled study to investigate whether telephone-based reeducation regarding the medication instructions of 14-day quadruple therapy could enhance the *H*. *pylori* eradication rate.

## 2. Materials and Methods

### 2.1. Study Design

This randomized controlled multicenter study was registered with ClinicalTrials.gov, number NCT03193450. The trial was implemented from October 18, 2017, to March 30, 2018, at nine centers in China. The centers involved in this study were located in northwestern China, which is a middle/low-income area. All patients signed the informed consent form.

### 2.2. Study Patients

The inclusion criteria were as follows: (1) patients aged 18-70 years old; (2) patients with *H*. *pylori* infections, diagnosed by ^13^C UBT. In clinical practice, patients had a routine screening for *H*. *pylori* infection by UBT if they had gastrointestinal symptoms. Gastroendoscopy was not compulsive, and the decision for this examination was made by both the doctors and the patient at the outpatient center; (3) patients were able to orally take medications; (4) no usage of other drugs contradicting medications for *H*. *pylori* eradication; and (5) patients were on adequate birth control for at least 4 weeks after the termination of *H*. *pylori* eradication.

The exclusion criteria were as follows: (1) pregnant or breastfeeding patients; (2) experienced previous failed treatment for *H*. *pylori* eradication; (3) patients treated with bismuth salts or antibiotics within 1 month before enrollment or treated with proton pump inhibitor (PPI) or H2 receptor antagonist within two weeks before enrollment; (4) serious heart disease, liver disease, kidney disease, malignant tumor, or alcoholism may jeopardize the safety or adherence; (5) patients who previously had upper gastrointestinal surgery; (6) patients who were not able to express complaint (e.g., mental disorder, psychoneurosis); (7) patients with active serious infections; or (8) patients who had significant gastrointestinal bleeding within 4 weeks prior to medications.

### 2.3. Sample Size

We estimated a sample size of 67 subjects for each group, with a randomization ratio of 1 : 1, by assuming an eradication rate of 96% in the experimental group and 80% in the control group, with a two-tailed *α* of 0.05 and 1 − *β* of 0.85 [3, 22, 23]. We hypothesized that approximately 20% of the patients may drop out of the study. Therefore, in total, 162 patients with *H*. *pylori* infection were finally enrolled in the study.

### 2.4. Randomization

All included subjects were randomized in a 1 : 1 ratio (according to a computer-generated randomization list) into two groups: reeducation group, in which patients received telephone-based reeducation via phone call and message on the 4^th^, 7^th^, and 10^th^ days of the course of treatment, or the control group, in which patients received instructions only at the time of getting the prescriptions. The methods of the drug administration were repeated during the phone call and message, which included information about the dosage and frequency. Randomization was stratified by age (<60 years vs. ≥60 years), sex (female vs. male), living area (city vs. rural), educational background (college vs. high school or less), economic conditions (monthly income ≥ 3000 yuan vs. <3000 yuan), and smoking history (yes vs. no).

### 2.5. Procedure

Patients in both the groups received a 14-day course *H*. *pylori* eradication therapy, which consisted of four medicines (esomeprazole 20 mg, colloidal bismuth tartrate 220 mg, amoxicillin 1000 mg, and clarithromycin 500 mg), administered twice daily. Patients who were allergic to amoxicillin received metronidazole 400 mg twice daily. The possible drug-related adverse events were recorded immediately after being reported by the patients or at the end of treatment. ^13^C urea breath test (UBT) was rechecked at 4-6 weeks after the end of treatment, and the symptoms were recorded at the same time. The UBT with high sensitivity and specificity is the best approach for the diagnosis of *H*. *pylori* infection [24]. This study was not blind to the doctors and subjects but was blind to the statisticians.

### 2.6. Outcome Measures

The primary outcome of the study was the *H*. *pylori* eradication rate. *H*. *pylori* infection was considered successfully eradicated when negative results were obtained by ^13^C UBT at 4-6 weeks after the end of the treatment. The secondary outcomes of the study included the symptom relief rates and adverse event rates. The symptoms were scored twice, based on Gastrointestinal Symptom Rating Scale (GSRS), at the points of baseline and at 4-6 weeks after the end of treatment. Symptom relief was defined as decreased GSRS score after eradication, when compared with the GSRS score before treatment.

### 2.7. Statistical Analysis

The intention-to-treat (ITT) and per-protocol (PP) analyses were used in the final analysis, while only PP analysis was used for GSRS score-related symptom evaluations. Continuous variables were summarized as means and standard deviation and analyzed using Student's *t*-test. Categorical variables were expressed as frequencies and percentages and analyzed using *χ*^2^ test or Fisher's exact test, as appropriate. A two-tailed *P* value < 0.05 was considered statistically significant. Statistical analyses were performed with SPSS (SPSS Inc., version 17.0, Chicago, IL).

## 3. Results

### 3.1. Participants

**Figure 1:**
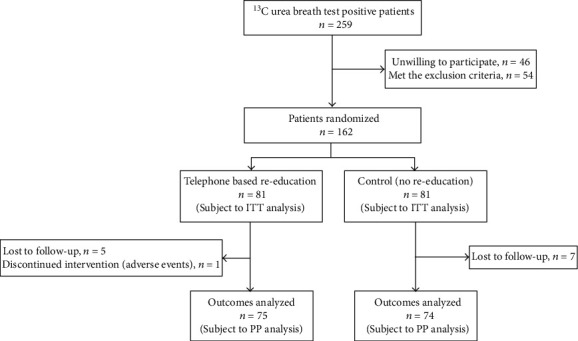
Flow chart of the study.

In total, 259 patients with positive UBT results were screened. Among these, 162 patients were enrolled into the study (46 were unwilling to participate and 54 met the exclusion criteria). The enrolled patients were randomized (1 : 1) to the reeducation group (*n* = 81) and control group (*n* = 81). There was no significant difference observed in patient baseline characteristics between the two groups (Table 1). Five patients in the reeducation group and seven patients in the control group were lost to follow-up. In addition, one patient in the reeducation group did not finish the medication due to diarrhea caused by antibiotics. Finally, 75 patients in the reeducation group and 74 patients in the control group completed the follow-up (*P* = 0.766) and consequently were included in the PP analysis (Figure 1).

### 3.2. Outcomes

The *H*. *pylori* eradication rates of the reeducation group and control group by PP analysis and ITT analysis were 78.7% (59/75) and 55.4% (41/74) (*P* = 0.003) and 72.8% (59/81) and 50.6% (41/81) (*P* = 0.006), respectively. No statistical difference between the two groups was observed with respect to the GSRS scores before the eradication (3.6 ± 1.9 vs. 3.7 ± 1.8, *P* = 0.905). The preexisting symptoms in 69 (92.0%) and 64 (86.5%) patients in the reeducation group and the control group, respectively, were relieved after the treatment (*P* = 0.449). However, the GSRS scores after treatment were significantly lower in the reeducation group (1.1 ± 0.9) when compared with the GSRS scores in the control group (2.0 ± 1.2) (*P* < 0.001). In addition, the GSRS scores were decreased by 2.5 ± 1.4 and 1.6 ± 1.0 in the reeducation and control groups, respectively (*P* < 0.001). The outcomes are summarized in Table 2.

Data regarding the adverse events reported during the treatment is shown in Table 3. The total adverse event rate in the reeducation group was higher than that in the control group (37.0% vs. 23.5% by ITT analysis and 40.0% vs. 25.7% by PP analysis, respectively). However, the difference was not statistically significant either by ITT (*P* = 0.087) or by PP (*P* = 0.081) analysis. Although no statistically significant difference was observed with respect to the occurrence of each adverse event, the occurrence rates of most adverse events in the reeducation group were higher than those in the control group (*P* ≥ 0.05).

## 4. Discussion

Clinicians face a situation of uncertainty during the treatment of *H*. *pylori* infections, which leads to the variability in the response to treatment. The involved factors include antibiotic resistance, adherence to treatment, and idiosyncratic differences among patients [25]. However, improving patients' adherence is a relatively convenient and cost-effective approach to help increase the eradication rate. In this multicenter randomized controlled study, we found that telephone-based reeducation improved the *H*. *pylori* eradication rates from 55.4% to 78.7% by PP analysis and from 50.6% to 72.8% by ITT analysis.

Considering the increasing failure of therapies, the 14-day quadruple therapy has been favored by the recent consensus and guidelines [20, 21]. A meta-analysis, which involved 21 randomized controlled studies, showed that the eradication rate was increased by 5% with the use of triple therapy for 14 days when compared with 7 days [26]. Moreover, a recent survey revealed that the most commonly used anti-*H*. *pylori* regimens in China became 14-day bismuth quadruple therapy [27]. However, the prescribed dosage and administration of 14-day bismuth quadruple therapy are different from the drug label instructions. The prolonged duration and increased complexity of the treatment may affect patient compliance, particularly in economically undeveloped areas, with a relatively lower education level. Adherence is defined as the extent to which the patients correctly follow medical instructions [28]. Patients with better adherence to *H*. *pylori* eradication therapy were reported to have a significantly higher therapeutic effectiveness rate than those with lower adherence (96% vs. 69%) [29]. Poor adherence to the medication often results in the failure of treatment and may increase the risk of resistance to antibiotics [13]. A randomized controlled study demonstrated that the number of patients who took more than 90% of the medications was increased by the medication counseling from a pharmacist, along with a follow-up telephone call, after the initiation of the therapy [30]. Therefore, we hypothesized that telephone-based education may help to increase the eradication rate of *H*. *pylori*. In our study, in order to improve the adherence of *H*. *pylori* eradication, detailed instructions of medication were provided through both phone call and message on the 4^th^, 7^th^, and 10^th^ days of the course. In addition, patients' questions were answered, when needed. These time points were chosen based on a three-day interval. Besides, the telephone-based reeducation was proved to better relieve the symptoms, which was confirmed by a significantly pronounced decrease in GSRS scores in the reeducation group (2.5 ± 1.4) than in the control group (1.6 ± 1.0). However, the ratio of patients with relieved symptoms in the reeducation group (92.0%) was not significantly higher than that in the control group (86.5%).

In addition, it was considered that increased dosages of drugs may result in higher probability of adverse events. In our study, more patients in the reeducation group reported adverse events. This may be caused by higher intake of the medication, potentially due to the telephone follow-up which included timely reminder of proper medication, question resolution, and comforting. However, the differences in adverse event rates between the two groups lacked statistical significance, both for the total adverse event rates and the rates of each adverse event. Interestingly, more patients in the reeducation group completed the whole eradication course, despite the higher adverse events rate, which further demonstrated that the telephone-based reeducation promoted the medication adherence.

Based on a comprehensive literature search, we identified a study that showed that daily telephone-based reeducation did not improve the *H*. *pylori* eradication rate and compliance [19]. There could be several possible reasons for the disagreements about the effects of reeducation. First, the previous study was based on the 10-day triple therapy, with relatively less complexity and shorter treatment course, which might be less dependent on reeducation. Moreover, the bismuth used in our 14-day quadruple therapy tends to cause more obvious adverse events, such as darkening of the stools, loss of hearing, and anxiety, leading to unauthorized drug discontinuance. Second, the previous study was carried out in Chongqing, a city with better-developed economy in China. However, our study was carried out in nine different centers in Xi'an, which is an economically developing region and has lower educational background, with 54.82% of the population being infected by *H*. *pylori*. Taking into consideration the relatively low income and educational level in the area around Xi'an, there was a relatively higher demand for reeducation as well.

There are a few limitations to our study that need to be acknowledged. First, all the patients included in the study were administrated the same eradication therapy. However, the effects of different therapy may vary among patients. It has been reported that *H*. *pylori* antibiotic resistance is increasing [31]. According to a previous report, the resistance rate for clarithromycin or levofloxacin is around 20-50% and about 40-70% for metronidazole. On the contrary, the resistance rate of amoxicillin is relatively lower, which is around 0-5%. The combination of clarithromycin and amoxicillin is generally used in clinical practice because of the lower drug resistance rate and incidence of adverse events [31]. The bismuth-containing quadruple therapy has been recommended by Chinese *H*. *pylori* consensus. The combinations of antibiotics that have been recommended are as follows: (1) amoxicillin+clarithromycin, (2) amoxicillin+levofloxacin, (3) amoxicillin+furazolidone, and (4) tetracycline+metronidazole or furazolidone [32]. In our study, we chose the 14-day bismuth quadruple therapy. Second, some adverse events might not be recorded as several patients failed to report the side effects in the form, which could result in recall bias. Lastly, the number of leftover pills was reported by patients but not counted by physicians, which could be inaccurate and affect the estimation of the number of patients who completed the medication course.

## 5. Conclusions

Our multicenter randomized controlled study is the first study to evaluate the effects of telephone-based reeducation on 14-day bismuth quadruple *H*. *pylori* eradication therapy. The reeducation was proved to be helpful for the improvement of the *H*. *pylori* eradication rate, symptom relief rate, and patients' adherence, without significantly increasing the adverse effects. Overall, telephone-based reeducation can be used as an effective intervention to improve the *H*. *pylori* eradication rate in clinical practice.

## Figures and Tables

**Table 1:** Baseline characteristics of all the included patients.

Characteristics	Intervention group (*n* = 81)	Control group (*n* = 81)	*P* value
Sex (M/F^†^)	36/45	34/47	0.874
Age	44.9 ± 13.3	44.3 ± 12.1	0.755
Residence			0.703
City	65 (80.2)	62 (76.5)	
Country	16 (19.8)	19 (23.5)	
Education			0.750
High school or lower	46 (56.8)	49 (60.5)	
Higher than high school	35 (43.2)	32 (39.5)	
Salary			0.636
<3000	42 (51.9)	46 (56.8)	
≥3000	39 (48.1)	35 (43.2)	
Smoking			
Yes	37 (45.7)	35 (43.2)	0.874
No	44 (54.3)	46 (56.8)	
Gastroscopy results			0.365
Gastritis	57 (76.0)	52 (68.4)	
Ulcer	18 (24.0)	24 (31.6)	

^†^M: male; F: female. Values are presented as mean ± SD or *n* (%).

**Table 2:** Effects of telephone-based reeducation on the eradication rate and symptoms after *H*. *pylori* eradication.

	Intervention group (*n* = 81)	Control group (*n* = 81)	*P* value
No. of completion of study	75 (93.8)	74 (91.4)	0.766
Treatment termination from adverse effect	1 (1.2)	0 (0)	1
Successful *H*. *pylori* eradication	59	41	0.006 (ITT)
	(ITT: 72.8, PP: 78.7)	(ITT: 50.6, PP: 55.4)	0.003 (PP)
GSRS^‡^ score before eradication	3.6 ± 1.9	3.7 ± 1.8	0.905
GSRS^‡^ score after eradication	1.1 ± 0.9	2.0 ± 1.2	<0.001
GSRS^‡^ alteration (after-before)	−2.5 ± 1.4	−1.6 ± 1.0	<0.001
Symptom relief based on GSRS^‡^ score	69 (PP: 92.0)	64 (PP: 86.5)	0.449

^‡^GSRS: Gastrointestinal Symptom Rating Scale. Values are presented as mean ± SD or *n* (%).

**Table 3:** Effects of telephone-based reeducation on adverse events after *H*. *pylori* eradication.

Adverse event	Intervention group *n* (%)	Control group *n* (%)	*P* value
Diarrhea	2	3	1 (ITT)
	(ITT: 2.5, PP: 2.7)	(ITT: 3.7, PP: 4.1)	0.681 (PP)
Abdominal pain	6	5	1 (ITT)
	(ITT: 7.4, PP: 8.0)	(ITT: 6.1, PP: 6.8)	1 (PP)
Feces discolored	4	0	0.12 (ITT)
	(ITT: 4.9, PP: 5.3)	(0)	0.12 (PP)
Taste disorder	2	0	0.497 (ITT)
	(ITT: 2.5, PP: 2.7)	(0)	0.497 (PP)
Nausea	12	10	0.819 (ITT)
	(ITT: 14.8, PP: 16.0)	(ITT: 12.3, PP: 13.5)	0.818 (PP)
Skin rash	3	1	0.620 (ITT)
	(ITT: 3.7, PP: 4.0)	(ITT: 1.2, PP: 1.4)	0.620 (PP)
Dizziness	1	0	1 (ITT)
	(ITT: 1.2, PP: 1.3)	(0)	1 (PP)
Total	30	19	0.087 (ITT)
	(ITT: 37.0, PP: 40.0)	(ITT: 23.5, PP: 25.7)	0.081 (PP)

## Data Availability

The data of this study would be available on request through the corresponding author. The corresponding author is Shuixiang He with the e-mail address hesx123@126.com. The contacted address is 277 West Yanta Road, Xi'an, Shaanxi 710061, China (Fax: +86-85323112; Tel: +86-85323112).
